# Supplementary material for: No evidence for the use of magnetic declination for migratory navigation in two songbird species
Source: PLoS One. 2020 Apr 24;15(4):e0232136. doi: 10.1371/journal.pone.0232136 (PMC7182221; doi:10.1371/journal.pone.0232136)
Supplement: S1 Table — This table contains all results of individual orientation tests based on which circular diagrams in Fig 1 were plotted and statistics presented in the Results section were calculated. (DOC) [file pone.0232136.s001.doc]

Table S1. Results of individual orientation tests, degrees (0° is geographic North). NMF is the natural magnetic field of the testing site, Courish Spit (55°09´ N, 2052´ E). CMF is changed magnetic field, i.e. virtual magnetic displacement to northern Scotland (*~* 56°30´ N, 3°20´ W).

| Adult European robins, NMF | Adult European robins, CMF | First-year European robins, NMF | First-year European robins, CMF | Adult garden warblers, NMF | Adult garden warblers, CMF |
| --- | --- | --- | --- | --- | --- |
| 283.5 | 324 | 220.5 | 267 | 237.5 | 237 |
| 329.5 | 248 | 255.5 | 284 | 270.5 | 157 |
| 328.5 | 179 | 225.5 | 230 | 305.5 | 211 |
| 309.5 | 182 | 177.5 | 268 | 196.5 | 207 |
| 36.5 | 241 | 285.5 | 182 | 147.5 | 222 |
| 296.5 | 259 | 145.5 | 162 | 260.5 | 217 |
| 215.5 | 192 | 245.5 | 134 | 206.5 | 245 |
| 227.5 | 286 | 230.5 | 271 | 181.5 | 214 |
| 242.5 | 313 | 335.5 | 177 | 285.5 | 198 |
| 308.5 | 20 | 292.5 | 263 | 146.5 | 127 |
| 270.5 | 255 | 257.5 | 282 | 320.5 | 113 |
| 202.5 | 237 | 193.5 | 137 | 170.5 | 188 |
| 325.5 | 359 | 278.5 | 182 | 125.5 | 259.5 |
| 272.5 | 307 | 301.5 | 207 | 168.5 | 198 |
| 335.5 |  | 137.5 |  | 314.5 | 83 |
| 17.5 |  |  |  | 120.5 | 214 |
| 290.5 |  |  |  | 124.5 | 218 |
|  |  |  |  | 164.5 | 91 |
|  |  |  |  | 208.5 | 157 |
|  |  |  |  | 188.5 | 315 |
|  |  |  |  | 297.5 |  |
|  |  |  |  | 81.5 |  |
|  |  |  |  | 236.5 |  |
|  |  |  |  | 80.5 |  |
|  |  |  |  | 205.5 |  |
