# Supplementary figures and images for: No evidence for the use of magnetic declination for migratory navigation in two songbird species
Source: PLoS One. 2020 Apr 24;15(4):e0232136. doi: 10.1371/journal.pone.0232136 (PMC7182221; doi:10.1371/journal.pone.0232136)

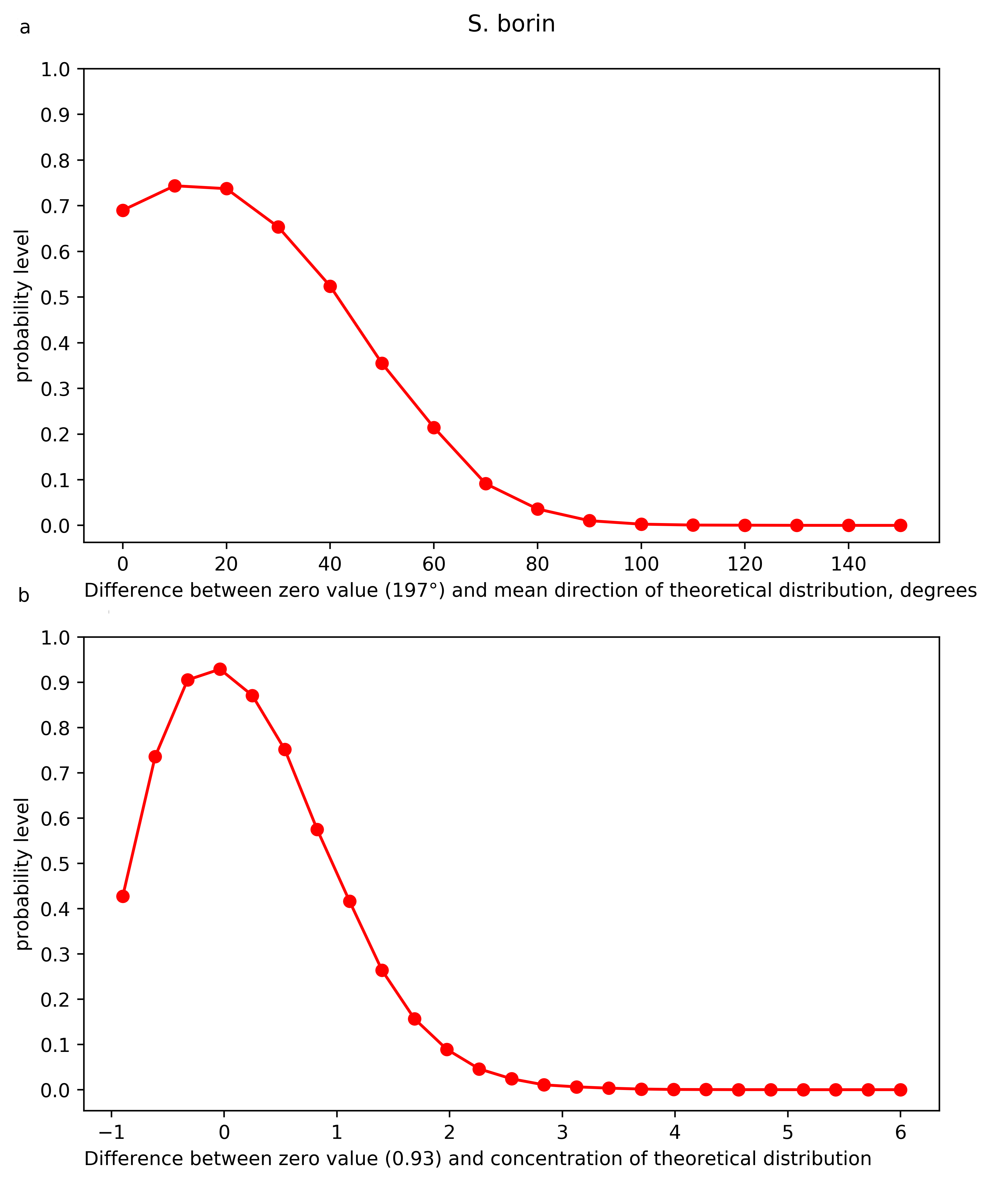

Supplement: S1 Fig — a) The probability to obtain p > 0.2 (according to our result of the MWW test: W = 2.75, P = 0.253) depending on how much the true mean direction of the experimental group (in the CMF) differs from the mean direction of the control group (in the NMF); b) The same for concentrations. Zero value for the mean direction is 197° (the mean direction in the CMF), for the concentration 0.93. (PNG) [file pone.0232136.s002.png]

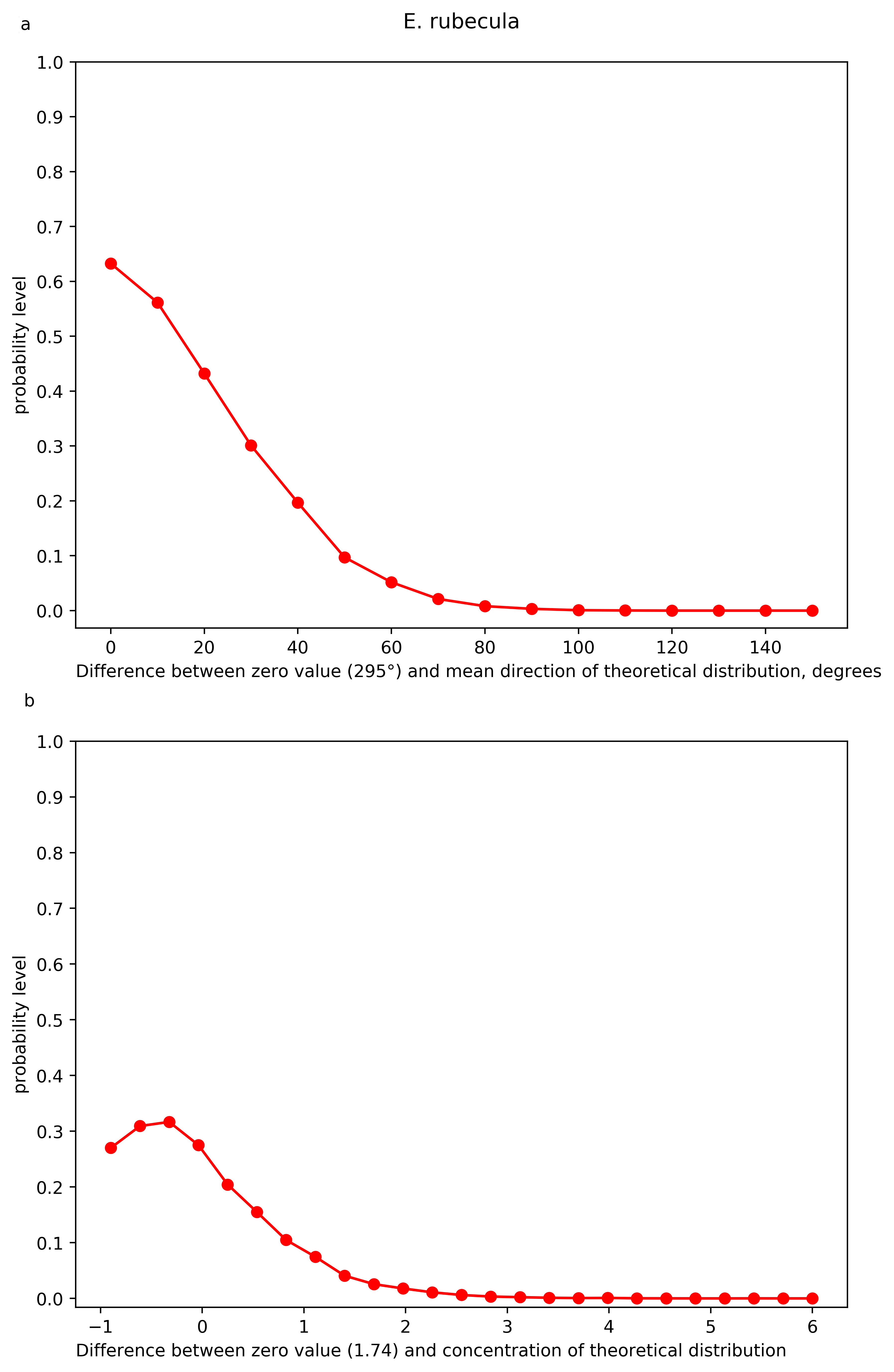

Supplement: S2 Fig — a) The probability to obtain p > 0.5 (according to our result of the MWW test: W = 1.19, P = 0.55) depending on how much the true mean direction of the experimental group (in the CMF) differs from the mean direction of the control group (in the NMF); b) The for concentrations. Zero value for the mean direction is 295° (the mean direction in the CMF), for the concentration 1.74. (PNG) [file pone.0232136.s003.png]

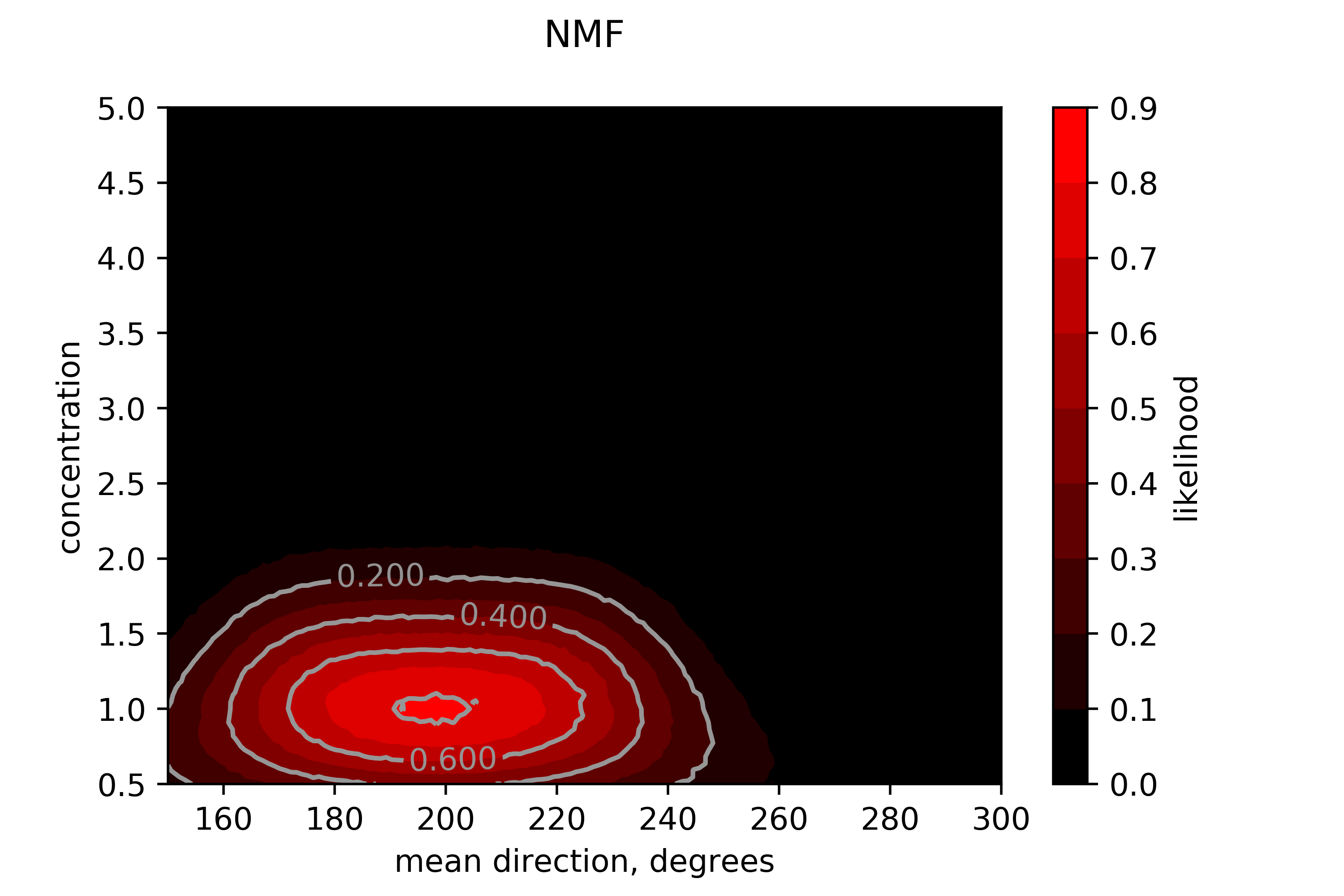

Supplement: S3 Fig — This estimation is based on the assumption that the statistical population is from a von Mises population. 95% CI was computed for the mean direction and length of the mean vector of our samples. Colour indicates the probability to obtain a sample with parameters (mean direction and length of the mean vector) included in our confidence intervals, from the von Mises distribution specified on the axes of the plot. Means of theoretical von Mises distribution are shown on X-axis, concentration on Y-axis. (PNG) [file pone.0232136.s004.png]

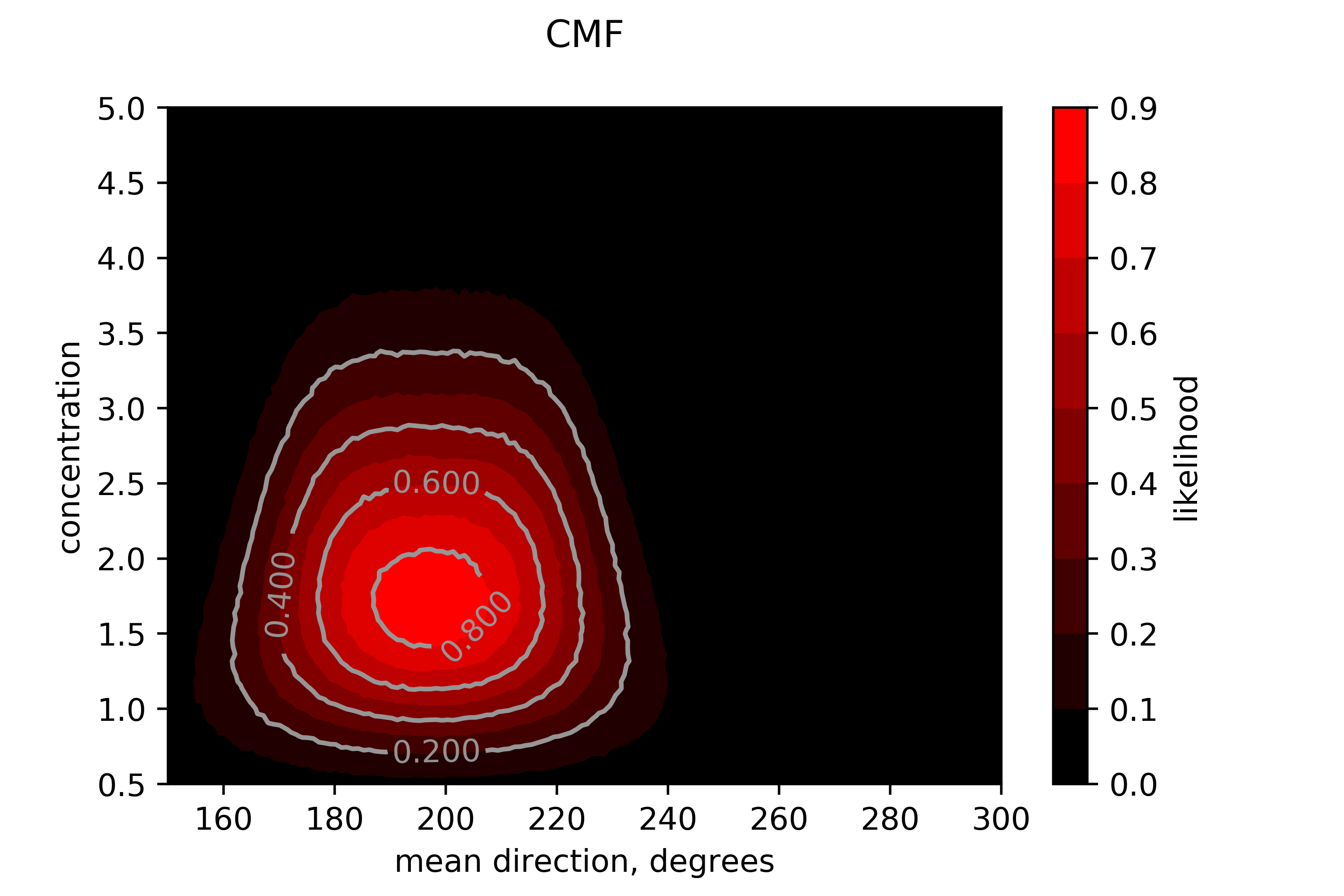

Supplement: S4 Fig — For description and explanation, see the legend to S3 Fig. (PNG) [file pone.0232136.s005.png]
